# DHA-Derived Lipid Mediators Attenuate Osteoarthritis by Resolving Inflammation and Protecting Cartilage in Association with the SIRT1 Signaling Pathway

**DOI:** 10.3390/md24060209

**Published:** 2026-06-12

**Authors:** Yan Su, Soon Kyu Kwon, Hack Sun Choi, Yunjon Han, Jung-Hee Park, Jong Hyun Choi, Jeong-Woo Seo

**Affiliations:** 1 Microbial Biotechnology Research Center, Korea Research Institute of Bioscience and Biotechnology (KRIBB), 181 Ipsin-gil, Jeongeup-si 56212, Republic of Korea; suyan@kribb.re.kr (Y.S.); ksk0469@kribb.re.kr (S.K.K.); gugin@kribb.re.kr (Y.H.); 2 Department of Biotechnology, Jeonbuk National University, Iksan 54596, Republic of Korea; junghee.park@jbnu.ac.kr; 3 Department of Biochemistry & Molecular Biology, Yonsei University College of Medicine, Seoul 03722, Republic of Korea; choix074@yuhs.ac

**Keywords:** lipid mediators, osteoarthritis, chondrocytes, MIA-induced model, inflammation, ECM

## Abstract

Osteoarthritis (OA) is a chronic degenerative joint disease characterized by persistent low-grade inflammation and progressive cartilage destruction. Macrophage-driven inflammatory responses contribute to extracellular matrix (ECM) degradation and accelerate disease progression. Here, we investigated the therapeutic potential of a DHA-derived lipid mediator mixture (LM), generated via soybean lipoxygenase and composed of 17S-hydroxydocosahexaenoic acid, resolvin D5, and protectin DX (3:47:50), in regulating macrophage–chondrocyte crosstalk and OA progression. LM significantly reduced IL-6, IL-1β, and TNF-α production in lipopolysaccharide-induced THP-1 macrophages. Conditioned medium from LM-treated macrophages attenuated ECM degradation in primary chondrocytes by suppressing MMP13 and ADAMTS5 while restoring COL2A1 and ACAN expression, indicating that LM may indirectly protects ECM by modulating the inflammatory microenvironment. In parallel, LM directly protected chondrocytes against IL-1β-induced inflammatory and catabolic responses, and restored ECM homeostasis. Mechanistically, LM significantly increased SIRT1 expression and deacetylation activity, as demonstrated by reduced NF-κB p65 acetylation. Both pharmacological inhibition by EX527 and siRNA-mediated SIRT1 knockdown abolished the protective effects of LM on ECM preservation. In vivo, LM oral administration alleviated cartilage destruction, improved joint structure and suppressed OA progression in a monosodium iodoacetate-induced OA model. Notably, micro-CT studies have demonstrated that LM significantly improved subchondral bone architecture, as evidenced by increased bone volume fraction and improved trabecular parameters. Histological analyses confirmed that LM attenuated inflammation and maintained cartilage integrity. Consistently, immunohistochemical findings showed reduced MMP13 expression, restoration of collagen II and aggrecan, and upregulation of SIRT1 in the LM-treated group compared to OA rats. Collectively, these findings suggest that LM mitigates OA progression by reducing inflammation, preserving ECM homeostasis, and attenuating subchondral bone deterioration.

## 1. Introduction

Osteoarthritis (OA) is one of the most common degenerative joint conditions, characterized by the gradual breakdown of cartilage, persistent inflammation of the synovial membrane, abnormal remodeling of the bone beneath the cartilage, and constant pain, significantly impairing patients’ quality of life [1,2]. Current clinical management is limited to symptomatic relief with nonsteroidal anti-inflammatory drugs, corticosteroid injections, and surgical intervention in advanced cases. However, these strategies have limited ability to address the underlying pathological mechanisms driving OA progression, highlighting the urgent need for novel disease-modifying approaches [3].

Chondrocytes are the sole resident cells within articular cartilage and are responsible for maintaining extracellular matrix (ECM) homeostasis through a tightly regulated balance between anabolic and catabolic processes [4]. Under OA conditions, chondrocytes undergo phenotypic alterations characterized by increased production of matrix-degrading enzymes, including MMP13 and ADAMTS5, accompanied by reduced synthesis of ECM components such as collagen II and aggrecan. This imbalance ultimately leads to progressive cartilage degeneration and loss of joint function [5].

In addition to chondrocyte dysfunction, chronic inflammation has emerged as a critical driver of OA pathogenesis [6,7]. Macrophages within the synovium, particularly those with the M1-like phenotype, produce pro-inflammatory mediators such as interleukin-1β (IL-1β), tumor necrosis factor-alpha (TNF-α), IL-6, NO, and PGE2, which accelerate cartilage matrix degradation by inducing catabolic enzymes, including matrix metalloproteinase-13 (MMP13) and aggrecanase (ADAMTS) [8]. Given the critical roles of inflammation and cartilage homeostasis in OA progression, regulators capable of modulating both processes have emerged as promising therapeutic targets. Sirtuin-1 (SIRT1), a NAD^+^-dependent deacetylase, counteracts inflammatory signaling by deacetylating NF-κB p65 and supporting ECM synthesis. Decreased SIRT1 expression is frequently observed in degenerative cartilage, suggesting its involvement in OA progression [9,10,11]. Consequently, agents capable of activating SIRT1 while restoring inflammatory and ECM homeostasis may hold considerable therapeutic potential for OA treatment.

Specialized pro-resolving mediators (SPMs) derived from omega-3 fatty acids actively promote inflammation resolution and restoring tissue homeostasis [12,13,14]. The lipid mediator mixture (LM), consisted of 17S-HDHA, resolvin D5 (RvD5), and protectin DX (PDX) at a ratio of 3:47:50, was generated through soybean lipoxygenase-mediated hydroxylation of DHA. DHA, a marine-based omega-3 polyunsaturated fatty acid, was supplied by KD Nutra (Bratvaag, Norway). Previous studies in our laboratory have demonstrated that LM modulated rheumatoid arthritis, asthma, and acute lung injury [15,16,17]. However, their therapeutic potential in OA has not been fully characterized, and the mechanisms by which LM affects macrophage-chondrocyte interactions remain unclear. In this study, we investigated both the indirect and direct actions of LM on cartilage preservation. Specifically, we assessed whether LM regulated macrophage-driven inflammatory response, inflammation-induced ECM degradation and attenuated chondrocytes from IL-1β–induced catabolic injury. Importantly, we further explored the effects of LM on OA progression in the monosodium iodoacetate (MIA)-induced rat model.

## 2. Results

### 2.1. LM Protected Chondrocytes from Macrophage-Induced Inflammation

**Figure 1:**
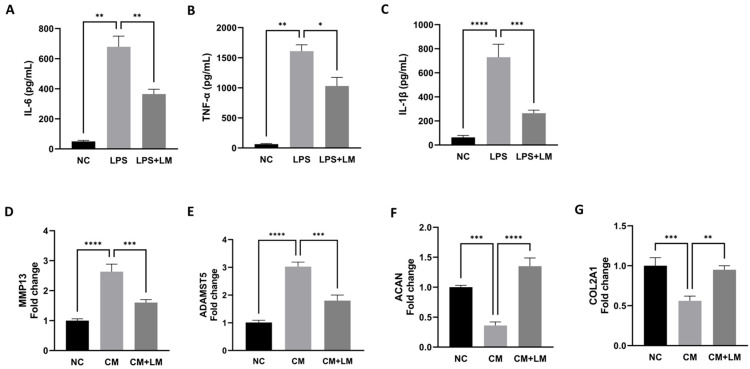
Effects of LM on inflammation and inflammation-induced ECM remodeling. THP-1 cells were stimulated by PMA (100 ng/mL) for 48 h, followed by LPS (1 μg/mL) induction with or without LM at 1 μg/mL for 24 h. (**A**–**C**) IL-6, TNF-α, and IL-1β levels in macrophage supernatant were measured by ELISA. Chondrocytes were cultured with THP-1 macrophage conditioned medium (CM) or LM-treat CM for 2 days. (**D**–**G**) Expression of MMP13, ADAMTS5, ACAN, and COL2A1 in chondrocytes. Data are presented as mean ± SD (*n* = 3). Statistical analysis was performed using one-way ANOVA followed by post hoc testing. * *p* < 0.05, ** *p* < 0.01, *** *p* < 0.001, **** *p* < 0.0001.

To assess the effects of LM on inflammation-driven ECM degradation, THP-1 monocytes differentiated into macrophages by PMA and subsequently stimulated with lipopolysaccharide (LPS) to generate a pro-inflammatory phenotype. ELISA results demonstrated that LPS markedly elevated IL-6, TNF-α, and IL-1β expression in macrophages. Treatment with LM, however, significantly reduced the level of these cytokines (Figure 1A–C).

Primary human chondrocytes were co-cultured with CM and LM-treated CM for 2 days. As shown in Figure 1D–G, CM significantly increased the expression of MMP13 and ADAMTS5 while decreasing the expression of ACAN and COL2A1. However, LM-treated CM reversed the shifts, downregulating MMP13 and ADAMTS5 expression and elevating ACAN and COL2A1 expressions. Taken together, these findings indicate that LM suppresses macrophage-driven inflammation, which may contribute to the attenuation of ECM degradation in chondrocytes.

### 2.2. LM Suppressed Inflammation in IL-1β-Induced Chondrocytes

**Figure 2:**
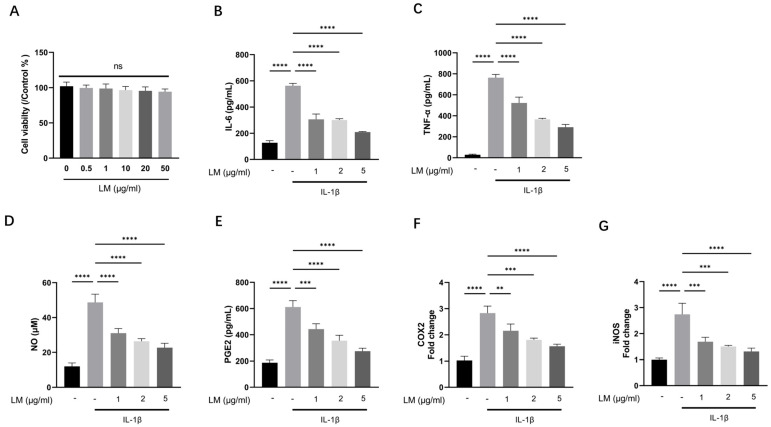
Effects of LM on inflammatory responses in IL-1β-stimulated human chondrocytes. (**A**) Cell viability was assessed by MTT assay after treatment with various concentrations of LM for 24 h. For subsequent experiments, human chondrocytes were pretreated with LM (1, 2, or 5 μg/mL) for 2 h and then stimulated with IL-1β (10 ng/mL) for 24 h. (**B**,**C**) The levels of IL-6 and TNF-α in the culture supernatants were measured by ELISA. (**D**,**E**) The production of NO and PGE2 was determined using commercial assay kits. (**F**,**G**) The mRNA expression levels of COX-2 and iNOS were analyzed by qRT-PCR. Data are presented as mean ± SD (*n* = 3). ns, no significant difference, ** *p* < 0.01, *** *p* < 0.001, and **** *p* < 0.0001.

Chondrocytes viability was first tested using MTT assays. As shown in Figure 2A, LM treatment at concentrations up to 50 μg/mL did not alter viability relative to untreated control. These results confirmed that LM within the tested range is non-cytotoxic and suitable for further functional experiments.

IL-1β stimulation significantly increased the secretion of the pro-inflammatory cytokines TNF-α and IL-6, whereas LM treatment markedly reduced their production in a concentration-dependent manner (Figure 2B,C). Similarly, the elevated levels of PGE2 and NO induced by IL-1β were significantly attenuated following LM treatment (Figure 2D,E). Consistent with these findings, qRT-PCR analysis demonstrated that LM suppressed the IL-1β-induced upregulation of COX-2 and iNOS mRNA expression (Figure 2F,G). Collectively, these results indicate that LM effectively alleviates inflammatory responses in chondrocytes.

### 2.3. LM Modulated ECM-Associated Genes in IL-1β-Induced Chondrocytes

**Figure 3:**
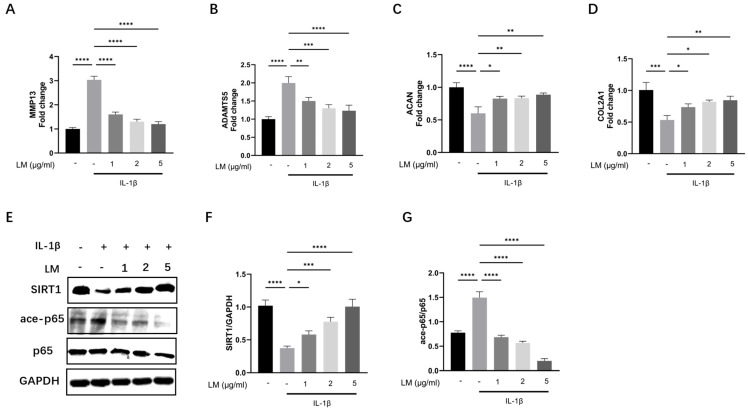
Effects of LM on extracellular matrix metabolism in IL-1β-stimulated human chondrocytes. Human chondrocytes were pretreated with LM (1, 2, or 5 μg/mL) for 2 h and subsequently stimulated with IL-1β (10 ng/mL) for 24 h. (**A**–**D**) qRT-PCR analysis of MMP13, ADAMTS5, ACAN, and COL2A1 in chondrocytes. (**E**) The expression of SIRT1 and p65 acetylation were detected by Western blotting. (**F**,**G**) Densitometric analysis of SIRT1 and p65 acetylation. Data are presented as mean ± SD (*n* = 3). Statistical analysis was performed using one-way ANOVA followed by post hoc testing. * *p* < 0.05, ** *p* < 0.01, *** *p* < 0.001, **** *p* < 0.0001.

Next, we examined the direct effects of LM on the key ECM-associated genes. qRT-PCR revealed that IL-1β treatment markedly increased MMP13 and ADAMTS5 expression to approximately 3-fold and 2-fold, respectively, compared with the control group. LM treatment significantly attenuated these increases in a dose-dependent manner. MMP13 expression was reduced by around 47%, 57%, and 60%, while ADAMTS5 expression was approximately reduced by 25%, 35%, and 38% at 1, 2, and 5 μg/mL of LM, respectively, compared with the IL-1β-treated group (Figure 3A,B). Conversely, LM treatment significantly reversed the IL-1β-induced suppression of ACAN and COL2A1, restoring their expression to approximately 1.3–1.5-fold above the levels observed in the IL-1β-treated group (Figure 3C,D). Taken together, these findings indicated that LM maintains cartilage homeostasis.

Given the role of SIRT1 in cartilage homeostasis and OA development [18], we investigated whether LM modulated SIRT1 expression in chondrocytes. Western blotting showed that LM significantly increased SIRT1 level compared with IL-1β alone group (Figure 3E,F). To further evaluate SIRT1 deacetylase activity, p65 acetylation was measured as a downstream indicator. LM treatment significantly reduced p65 acetylation compared with the IL-1β-treated group (Figure 3E,G). These findings suggested SIRT1/NF-κB signaling contributes, at least in part, to the protective effects of LM.

### 2.4. SIRT1 Contributed to the Protective Effects of LM in Chondrocytes

**Figure 4:**
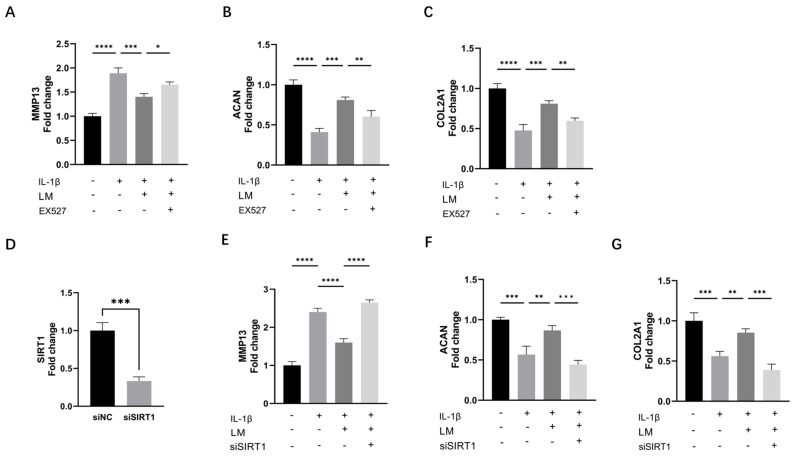
Effects of SIRT1 inhibition and knockdown on LM-treated chondrocytes. (**A**–**C**) Chondrocytes were pretreated with or without the SIRT1 inhibitor EX-527 (10 μM) for 1 h, followed by LM (5 μg/mL) for 2 h and subsequent stimulation with IL-1β (10 ng/mL) for 24 h. The mRNA expression levels of MMP13, ACAN, and COL2A1 were analyzed by qRT-PCR. (**D**–**G**) In parallel, SIRT1 was silenced using siRNA, with si-NC as control. The mRNA levels of MMP13, ACAN, and COL2A1 following IL-1β and LM treatment were quantified by RT-qPCR. Data are presented as mean ± SD (*n* = 3). Statistical analysis was performed using one-way ANOVA followed by post hoc testing. * *p* < 0.05, ** *p* < 0.01, *** *p* < 0.001, **** *p* < 0.0001.

Both pharmacological inhibition and genetic knockdown were employed to determine whether SIRT1 is required for the effects of LM. Treatment with the selective SIRT1 inhibitor EX-527 markedly attenuated the protective effects of LM, as evidenced by the reversal of MMP13 suppression and the reduced expression of ACAN and COL2A1 (Figure 4A–C). To further validate these findings, SIRT1 expression was silenced using siRNA. Efficient SIRT1 knockdown was confirmed by qRT-PCR (Figure 4D). Consistent with the pharmacological inhibition results, effects of LM were largely abolished in SIRT1-deficient cells. Specifically, LM failed to significantly suppress MMP13 expression or restore ACAN and COL2A1 levels following SIRT1 knockdown (Figure 4E–G). Collectively, these findings indicate that SIRT1 plays a critical role in mediating the protective effects of LM against IL-1β-induced ECM degradation in chondrocytes.

### 2.5. Optimal LM Concentration for Joint Protection in MIA-Induced OA Rats

**Figure 5:**
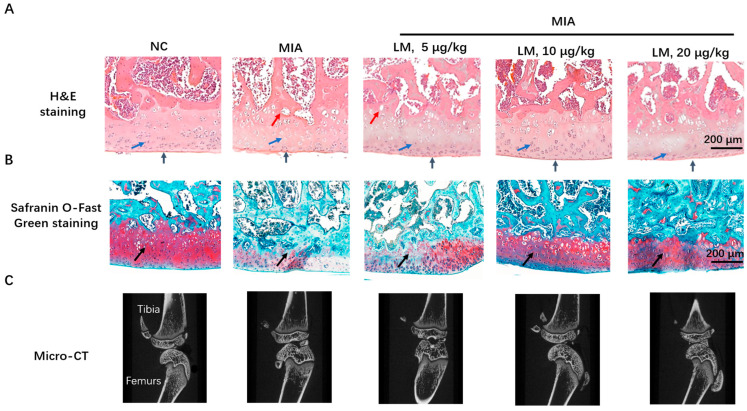
Effects of LM (5, 10, and 20 μg/kg) on joint in MIA-induced OA rats after 2 weeks. (**A**) The knees joints were stained by H&E staining (scale bar = 200 μm). Blue arrow: normal rows of chondrocytes. Gray arrow: cartilage surface. Red arrow: subchondral bone showed cyst formation. (**B**) Safranin O-Fast Green staining for cartilage damage (scale bar = 200 μm). Black arrow: cartilage. (**C**) Micro-CT analysis of subchondral bone microarchitecture cartilage structure.

The in vivo efficacy of LM was subsequently evaluated using an MIA-induced OA rat model. LM was orally administered at 5, 10, or 20 μg/kg for 2 weeks. H&E staining revealed that the articular cartilage in the NC group exhibited a smooth surface, normal thickness, and well-organized chondrocytes. In contrast, the MIA group showed obvious osteoarthritic changes, including cartilage surface erosion, reduced cartilage thickness, loss of chondrocytes, and subchondral cysts. However, LM treatment attenuated these pathological alterations (Figure 5A). Safranin O-Fast Green staining further demonstrated a marked reduction in cartilage in the MIA group, as evidenced by diminished staining intensity. In contrast, LM treatment preserved proteoglycan content, resulting in stronger staining and improved cartilage structural integrity (Figure 5B). Consistent with these findings, micro-CT analysis showed that MIA caused pronounced joint destruction, including irregular articular surfaces and narrowing of the joint space. These structural abnormalities were substantially alleviated by LM treatment, indicating its protective effects against OA-associated joint damage (Figure 5C). These results indicate that LM administration effectively against cartilage destruction and bone erosion in OA.

Of the three LM doses, 10 μg/kg induced the greatest improvements, evidenced by smoother cartilage surfaces, stronger matrix staining, and improved structural integrity. In contrast, a treatment at 5 μg/kg showed weaker effects, while 20 μg/kg did not provide additional benefit. These outcomes suggest an optimal therapeutic window rather than a simple dose-dependent response.

### 2.6. LM Attenuated Cartilage Degradation in MIA-Induced OA Rats

**Figure 6:**
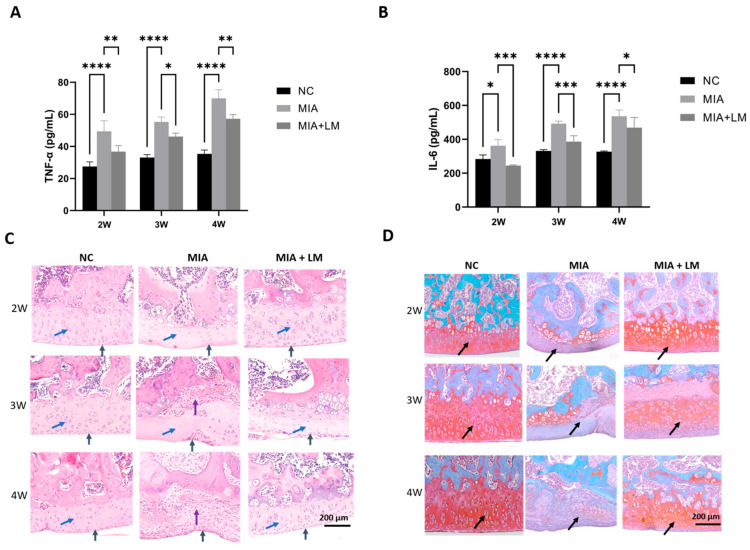
Effects of LM (10 μg/kg) on cartilage in MIA-induced OA rats after 2, 3, and 4 weeks. The level of (**A**) TNF-α and (**B**) IL-6 in serum were detected by ELISA kit. Data are presented as mean ± SD (*n* = 3). Statistical analysis was performed using one-way ANOVA followed by post hoc testing. * *p* < 0.05, ** *p* < 0.01, *** *p* < 0.001, **** *p* < 0.0001. The knees joints were stained by (**C**) H&E staining (scale bar = 200 μm) and (**D**) Safranin O-Fast Green staining of cartilage damage (scale bar = 200 μm). In H&E-stained sections, blue arrows indicate normal parallel rows of chondrocytes; gray arrows indicate the cartilage surface; and purple arrows indicate inflammatory cell infiltration. In Safranin O–Fast Green-stained sections, black arrows indicate cartilage tissue.

To evaluate the efficacy of LM during disease progression, we administered the optimal dose (10 μg/kg) for 2, 3, and 4 weeks. Inflammatory cytokines in serum were measured by ELISA, revealing elevated TNF-α and IL-6 levels in the MIA group at all time points, which were significantly reduced by LM treatment (Figure 6A,B).

Histological examination revealed progressive cartilage degeneration in the MIA group from week 2 to week 4. H&E staining showed surface erosion, disruption of cartilage architecture, loss of chondrocytes, and inflammation cells infiltration, which became increasingly severe over time. In contrast, LM treatment preserved cartilage morphology and reduced structural damage throughout the experimental period (Figure 6C). Safranin O-Fast Green staining was performed to evaluate cartilage integrity and proteoglycan content in the knee joints (Figure 6D). In the NC group, the articular cartilage exhibited a smooth surface with intense Safranin O-Fast Green staining, indicating abundant proteoglycan content and preserved cartilage architecture. In contrast, the MIA group showed progressive cartilage degeneration from week 2 to week 4, characterized by structural disorganization and a marked reduction in Safranin O-Fast Green staining intensity, suggesting substantial loss of proteoglycans. LM treatment markedly attenuated these pathological changes, as evidenced by improved cartilage morphology and stronger Safranin O-Fast Green staining compared with the MIA group. These findings indicate that LM protects against MIA-induced cartilage degradation and extracellular matrix loss at all time points.

### 2.7. LM Attenuated Bone Loss in MIA-Induced OA Rats

**Figure 7:**
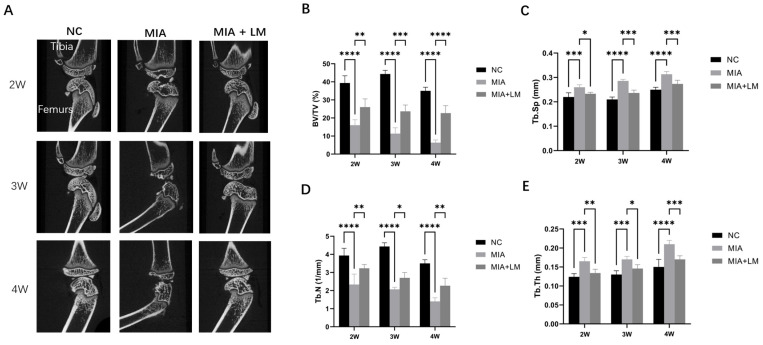
Effects of LM (10 μg/kg) on bone loss in MIA-induced OA rats after 2, 3, and 4 weeks. (**A**) Micro-computed tomography (micro-CT) analysis was performed to evaluate subchondral bone structural parameters. Quantitative analysis was calculated, (**B**) bone volume fraction (BV/TV), (**C**) trabecular separation (Tb. Sp), (D) trabecular number (Tb. N), and (**E**) trabecular thickness (Tb. Th). Data are presented as mean ± SD (*n* = 3). Statistical significance was determined by one-way ANOVA followed by post hoc analysis. * *p* < 0.05, ** *p* < 0.01, *** *p* < 0.001, **** *p* < 0.0001.

Micro-CT analysis revealed progressive subchondral bone loss and disruption in the MIA group (Figure 7A). Our observations showed that MIA treatment induced severe joint destruction involving both the femoral and tibial articular surfaces, characterized by irregular articular contours and disruption of the underlying subchondral bone architecture. These pathological changes became more pronounced from week 2 to week 4. In contrast, LM treatment markedly attenuated these pathological changes and preserved the structural integrity of both articular surfaces. Moreover, OA induction resulted in significant deterioration of bone microarchitecture, characterized by decreased BV/TV, Tb. Th, and Tb. N, along with increased Tb. Sp, compared with the NC group at each time point (Figure 7B–E). Notably, LM treatment markedly improved these parameters, indicating a protective effect on subchondral bone integrity. These findings suggest that LM effectively attenuates OA-associated bone structural damage and suppresses the OA progression.

### 2.8. LM Restored ECM Homeostasis in Cartilage of MIA-Induced OA Rats

**Figure 8:**
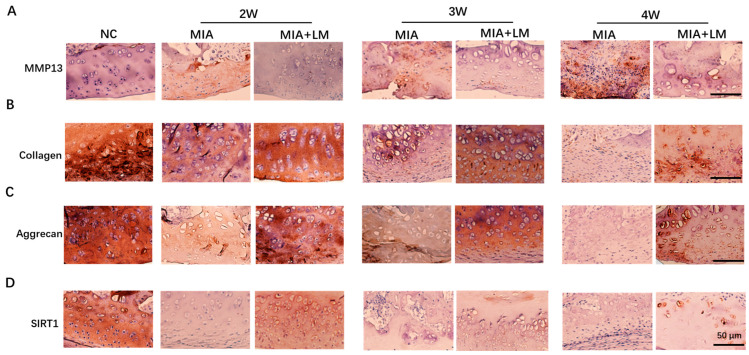
IHC evaluation of LM (10 μg/kg) on cartilage in MIA-induced OA rats after 2, 3, and 4 weeks. (**A**) MMP13, (**B**) Collagen II, (**C**) Aggrecan, and (**D**) SIRT1 (scale bar = 50 μm) in knee joints were detected by IHC.

To evaluate the effects of LM on cartilage in vivo, IHC was performed to analyze knee joint sections from MIA-induced OA rats at 2, 3, and 4 weeks (Figure 8). Compared with the NC group, MIA induction markedly increased the expression of MMP13. Notably, LM treatment significantly suppressed MMP13 expression at all time points (Figure 8A). Consistent with this, the reduced expression of collagen II and aggrecan observed in OA cartilage was markedly restored following LM treatment (Figure 8B,C). Simultaneously, SIRT1 expression significantly decreased in the MIA group compared with the NC group, whereas LM treatment significantly restored SIRT1 levels across all time points (Figure 8D). These effects became more pronounced over time, particularly at 3 and 4 weeks, demonstrating the sustained protective effect of LM on cartilage integrity. Collectively, these findings demonstrate that LM alleviates cartilage degradation in OA by suppressing catabolic activity and restoring ECM homeostasis, potentially in association with SIRT1upregulation.

## 3. Discussion

OA is increasingly recognized as a disease of the entire joint, in which pathological interactions between articular cartilage, synovium, and subchondral bone jointly drive disease progression [19]. In OA joints, chronic low-grade inflammation is a major driver of cartilage degeneration, subchondral bone remodeling, and synovitis [20,21]. Marine fish oils are among the richest natural sources of DHA, the precursor of SPMs. In recent years, SPMs derived from ω-3 polyunsaturated fatty acids have emerged as important endogenous regulators of inflammation resolution, which could terminate inflammatory responses while promoting tissue repair without inducing immunosuppression [22]. Several studies have demonstrated beneficial effects of LM consisting of 17S-HDHA, RvD5, and PDX in inflammatory and degenerative diseases, including arthritis and periodontitis [15,17]. However, the therapeutic potential and underlying mechanisms of LM in OA remain poorly understood. The present study therefore investigated the effects of LM on macrophage-mediated inflammation, chondrocyte dysfunction, and OA progression using both in vitro and in vivo models.

Synovial macrophages represent one of the most abundant immune cell populations within OA joints [4,7,23]. Activated synovial macrophages produce elevated levels of proinflammatory cytokines, which not only amplify local inflammation but also accelerate ECM degradation in cartilage [24,25]. Consistently, LM treatment markedly suppressed the expression of TNF-α, IL-6, and IL-1β in LPS-stimulated THP-1 macrophages, indicating that LM effectively attenuates macrophage-driven inflammatory responses. Importantly, CM from LM-treated macrophages significantly reduced ECM degradation in chondrocytes, suggesting that LM modulates inflammatory activity and inflammation induced ECM degradation.

Chondrocytes are the only resident cells in cartilage and are responsible for both synthesis and turnover of the ECM [26,27,28]. Collagen II and aggrecan are primary ECM components essential for tissue integrity and joint function, and their loss is a hallmark of OA progression [29,30,31]. IL-1β activates inflammatory and catabolic signaling pathways in chondrocytes, leading to increased production of pro-inflammatory mediators and matrix-degrading enzymes, ultimately resulting in the breakdown of collagen and proteoglycans within the cartilage ECM [12,26]. In the present study, IL-1β stimulation markedly increased the levels of TNF-α, IL-6, PGE2, and NO, accompanied by elevated expression of COX-2 and iNOS, confirming the establishment of a robust inflammatory response. In addition, IL-1β enhanced expression of MMP13 and ADAMTS5 together with suppression of anabolic matrix components. Previous studies have demonstrated that several SPMs can attenuate inflammatory signaling and reduce cartilage catabolism [12,13,14]. Consistent with these findings, LM significantly suppressed the production of inflammation mediators, as well as the expression of COX-2 and iNOS, indicating potent anti-inflammatory activity. Moreover, LM restored the expression of ACAN, and COL2A1 while reducing MMP13 and ADAMTS5 expression, suggesting that LM effectively protects chondrocytes from IL-1β-induced ECM degradation and preserves cartilage homeostasis.

Reduced SIRT1 expression has been observed in degenerated human cartilage and experimental OA models [32,33,34]. In the context of osteoarthritis, impaired SIRT1 activity has been associated with enhanced NF-κB signaling and a shift toward a sustained catabolic and pro-inflammatory chondrocyte phenotype [33,35]. In the present study, LM significantly increased SIRT1 expression while reducing acetylation of p65 at Lys310. Furthermore, both pharmacological inhibition and siRNA-mediated silencing of SIRT1 largely abolished the protective effects of LM on ECM-associated genes, suggesting that SIRT1 is a key mediator of LM effect. Collectively, these results suggest that LM exerts anti-inflammatory and matrix-preserving effects, at least in part, through activation of the SIRT1–NF-κB signaling axis, thereby restoring the balance between anabolic and catabolic processes in chondrocytes.

To further validate the protective effects of LM observed in vitro, we employed an MIA-induced rat model of OA. Consistent with the anti-inflammatory effects observed in macrophages, oral administration of LM significantly reduced both serum and knee inflammatory responses in OA rats. Chronic inflammation is recognized as a critical driver of OA progression, promoting cartilage catabolism and pathological changes in subchondral bone [6,7]. Therefore, suppression of inflammatory mediators by LM may contribute to the attenuation of joint tissue damage observed in vivo.

Histological analyses further demonstrated that LM alleviated cartilage degeneration, as evidenced by reduced chondrocyte loss, preservation of cartilage surface integrity. In addition to cartilage protection, micro-CT analysis revealed that LM improved subchondral bone microarchitecture, including increased bone volume fraction and improved trabecular parameters. Given the growing recognition that OA is a whole-joint disease involving reciprocal interactions between cartilage and subchondral bone, these findings indicate that LM exerts broader protective effects on joint structural integrity beyond cartilage alone. IHC analyses demonstrated that LM suppressed the expression of the catabolic enzymes MMP13 and ADAMTS5 while restoring collagen II and aggrecan levels in articular cartilage. These changes indicate a shift from a degradative to a reparative cartilage phenotype and support the ability of LM to restore ECM homeostasis in vivo. Notably, LM also increased SIRT1 expression in cartilage. Importantly, the protective effects of LM became increasingly evident with prolonged treatment, suggesting that LM may slow OA progression. Collectively, these findings suggest that LM mitigates OA progression by suppressing inflammatory responses, restoring ECM homeostasis, and improving subchondral bone microarchitecture.

## 4. Materials and Methods

### 4.1. Chemicals and Reagents

DHA was purchased from KD Nutra (Bratvaag, Norway). DMSO, MTT reagent, PMA, LPS, and recombinant human IL-1β were obtained from Sigma-Aldrich (St. Louis, MO, USA). Cell culture media and supplements were obtained from Gibco (Thermo Fisher Scientific, Waltham, MA, USA). ELISA kits for TNF-α and IL-6, as well as assay kits for PGE2 determination, were purchased from Abcam (Cambridge, UK). Nitric oxide (NO) assay kit (Griess Reagent System) was purchased from Promega (Madison, WI, USA). EX-527 was ordered from BioGems International (Westlake Village, CA, USA). Primary antibodies against SIRT1, p65, MMP13, collagen II, aggrecan and GAPDH were obtained from Abcam (Cambridge, UK). Acetylated p65 was purchased from Cell Signaling Technology (Danvers, MA, USA).

### 4.2. Cell Viability

Primary human chondrocytes sourced from Innoprot (Bizkaia, Spain) were cultured in special medium (Innoprot) at 37 °C in 5% CO_2_. To assess the impact of LM on chondrocyte survival rates, cells were seeded into 96-well plate overnight. The following day, cells were exposed to varying concentrations of LM for a 24 h period. For LM preparation, LM was initially dissolved in DMSO to prepare a stock solution and subsequently diluted with medium to the working concentrations immediately before use. The final DMSO concentration in the culture medium was maintained at a negligible level and did not affect cell viability. Cell viability was quantified using the MTT assay following the supplier’s recommended procedure. In brief, 100 μL of MTT reagent at 5 mg/mL was added to each well and incubated for 3 h. Afterward, the culture medium was extracted, and the resulting formazan precipitates were solubilized in 100 μL of DMSO. Finally, optical density measurements were measured at 540 nm wavelength using a Bio-Rad microplate reader (Hercules, CA, USA). All experiments were performed three independent biological replicates.

### 4.3. Macrophage-Chondrocyte Co-Culturing

Human monocytic THP-1 cells (KCLB, Seoul, Republic of Korea) were maintained in RPMI-1640 medium supplemented with 10% fetal bovine serum (FBS) and 1% penicillin–streptomycin at 37 °C in 5% CO_2_. To induce macrophage differentiation, THP-1 cells were treated with phorbol 12-myristate 13-acetate (PMA, 100 ng/mL) for 48 h, followed by overnight resting in PMA-free medium. Differentiated macrophages were pretreated with LM at 1 μg/mL for 2 h and subsequently stimulated with LPS (1 μg/mL) for 24 h to induce an inflammatory phenotype. Culture supernatants were collected, centrifuged to remove cellular debris, and used as macrophage-conditioned medium (CM). The cytokine levels (IL-6, TNF-α, and IL-1β) were quantified using ELISA kits. Primary chondrocytes were seeded and cultured until 70–80% confluence, then incubated with CM for 48 h. Cells were harvested for subsequent gene expression detection. All experiments were performed three independent biological replicates.

### 4.4. Protective Effects on Chondrocytes

To evaluate the direct protective effects of LM on chondrocytes, cells were pretreated with LM at 1, 2, and 5 μg/mL for 2 h, then stimulated with recombinant human IL-1β (10 ng/mL) for 24 h. These concentrations were selected based on our previous studies demonstrating significant biological activity of LM within this concentration range [15,16,17]. The levels of PGE2, IL-6, TNF-α, and NO in culture supernatants were quantified using ELISA. Total RNA and protein were extracted for qRT-PCR and Western blot analyses, respectively.

### 4.5. SIRT1 Inhibition with EX-527 In Vitro

To elucidate the influence of SIRT1 on LM, cells were treated with the selective SIRT1 inhibitor EX-527. Briefly, cells were pretreated with EX-527 (10 μM in DMSO) for 1 h, followed by treatment with LM (5 μg/mL) for 2 h, and subsequently stimulated with IL-1β for 24 h [18]. After treatment, cells were collected for qRT-PCR analysis.

### 4.6. siRNA Transfection

SIRT1 knockdown was performed using a validated human SIRT1 siRNA pool (Santa Cruz Biotechnology, Santa Cruz, CA, USA). A non-targeting siRNA served as the control (siCtrl). Transfections were conducted with Lipofectamine™ RNAiMAX (Thermo Fisher Scientific, Waltham, MA, USA) with all experimental procedures performed according to the manufacturer’s instructions. Briefly, siRNA was diluted in Opti-MEM™ medium (Thermo Fisher Scientific) and complexed with Lipofectamine (Thermo Fisher Scientific) per well in six-well plates. Complexes were added dropwise to cells at ~50–60% confluence. After 6 h, the medium was replaced with fresh antibiotic-free complete medium, and cells were cultured for an additional 24 h with or without treatment with IL-1β and LM treatment (5 μg/mL). Cells were collected for further experiments.

### 4.7. qRT-PCR

Total RNA was isolated from cells using a MiniBEST kit (TaKaRa, Tokyo, Japan). Transcript levels were quantified with a One-Step AccuPower GreenStar RT-qPCR PreMix kit (Bioneer Corporation, Daejeon, Republic of Korea). qRT-PCR was performed on the CFX Connect system (Bio-Rad, San Diego, CA, USA). The expression levels of ECM genes (SOX9, COL2A1, and ACAN) and matrix-degrading enzymes (MMP13 and ADAMTS5) were normalized to GAPDH. Relative expression was calculated using the 2^−ΔΔCt^ method. All primer sequences used in this study are listed in Table 1. All experiments were performed three independent biological replicates.

### 4.8. Western Blotting

The proteins were isolated using radioimmunoprecipitation assay buffer (Biosolution, Seoul, Republic of Korea) mixed with a protease inhibitor cocktail on ice for 30 min. Subsequently, the cell lysates were centrifuged at 12,000 rpm for 10 min at 4 °C. The proteins were separated through electrophoresis on sodium dodecyl sulfate–polyacrylamide gel electrophoresis gels and subsequently transferred to polyvinylidene fluoride membranes (Millipore, Sigma, St. Louis, MO, USA). The membranes were blocked with 5% bovine serum albumin (BSA, Thermo Fisher Scientific) dissolved in Tris-buffered saline with Tween 20 (TBST, Biosolution) for one hour. They were then incubated overnight at 4 °C with primary antibodies targeting SIRT1 (ab189494, Abcam), p65 (ab32536, Abcam), and acetylated p65 (Cell Signaling Technology, Danvers, MA, USA). After thorough washing with TBST, the membranes were incubated with HRP-linked secondary antibodies for 3 h at room temperature. The final step involved detecting protein bands with electrochemiluminescence substrate (Thermo Fisher Scientific) and analyzing their intensity using the NIH ImageJ software 1.48. All experiments were performed three independent biological replicates.

### 4.9. Animal Experiment Protocols

Male Sprague–Dawley rats (8 weeks old, 250–280 g) were purchased from Orient Bio Inc. (Seongnam, Republic of Korea) and used to establish a MIA-induced OA model [12]. All procedures were approved by the Institutional Animal Care and Use Committee of KRIBB and followed institutional guidelines. Animals were randomly assigned to experimental groups using a computer-generated randomization schedule to minimize selection bias. Allocation was performed before treatment initiation to ensure unbiased group assignment.

The rats were divided into five groups (n = 5/group): normal control (NC), MIA only, and MIA + LM at 5, 10, or 20 μg/kg. LM was initially dissolved in ethanol to prepare a stock solution and subsequently diluted with sterile normal saline immediately before administration. Animals in NC and MIA groups received an equivalent volume of vehicle. Rats received a single intra-articular injection of 3 mg MIA (Sigma) into the knee joint. LM was then orally administered once daily starting on Day 1 post-injection for 2 weeks. After histological evaluation, the most effective concentration was selected.

In a follow-up experiment, rats (n = 5/group) were injected 3 mg MIA and treated with or without LM (10 μg/kg) for 2, 3, or 4 weeks to evaluate the time-dependent effects of LM. Animals were euthanized, and serum samples were collected for cytokine analysis. The levels of IL-6 and TNF-α were quantified using ELISA kits (Abcam). Knee tissues were also collected for subsequent analyses.

### 4.10. Histological Evaluation

The collected knee joints were fixed in 4% paraformaldehyde, decalcified with EDTA, embedded in paraffin, sliced into 5 μm sections, and stained with hematoxylin and eosin (H&E) and Safranin O-Fast Green (Sigma) to assess cartilage morphology [17].

### 4.11. Micro-Computed Tomography

Micro-computed tomography (micro-CT) was used to evaluate bone architecture. Knee joints were scanned on a high-resolution micro-CT system (SkyScan 1276, Bruker, Kontich, Belgium) under the following specifications: 17.25 μm, 70 kV, and 200 μA. Three-dimensional reconstructions and quantitative analyses of bone volume fraction (BV/TV), trabecular thickness (Tb. Th), trabecular number (Tb. N), and trabecular separation (Tb. Sp) were performed with CTAn software 1.20.3.0 (Bruker) [17].

### 4.12. Immunohistochemistry

For the immunohistochemical (IHC) analysis, 5 μm sections of paraffin-embedded knee joints were prepared by removing the paraffin and rehydrating the tissues. Antigen retrieval was then performed using citrate buffer (pH 6.0) heated to 95 °C for 15 min. Endogenous peroxidase activity was knocked out by treating the samples with 3% hydrogen peroxide for 10 min, followed by blocking with 5% BSA at room temperature. The sections were incubated with primary antibodies overnight at 4 °C targeting SIRT1 (ab189494, Abcam), MMP13 (ab219620, Abcam), collagen II (ab307674, Abcam), and aggrecan (ab315486, Abcam). Following thorough washing, HRP-conjugated secondary antibodies (Abcam) were applied for 2 h at 37 °C. Signal visualization was performed using a DAB substrate kit (Solarbio, Beijing, China), followed by hematoxylin counterstaining (Abcam). Images were captured using a light microscope, and staining intensity was quantified using NIH ImageJ 1.48.

### 4.13. Statistical Analyses

All data are presented as mean ± standard deviation (SD). Statistical significance was assessed by one-way ANOVA followed by Tukey’s post hoc tests for parametric datasets as implemented in GraphPad Prism 9.5.1. For all statistical tests, *p*-values < 0.05 were considered statistically significant.

## 5. Conclusions

Overall, the ability of LM to suppress inflammation, preserve ECM homeostasis, and maintain joint structural integrity highlights its potential as a disease-modifying therapeutic strategy for OA through the SIRT1/NF-κB signaling pathway. Nevertheless, several limitations should be acknowledged. First, LM is a mixture of 17S-HDHA, RvD5, and PDX, and the present study did not investigate the individual effects of each component. Consequently, the relative contribution of each lipid mediator and their potential synergistic interactions remain to be elucidated in future studies. Second, further studies are required to determine whether LM regulates macrophage polarization. In addition, given the established role of SIRT1 in autophagy regulation and cartilage homeostasis, future studies should investigate whether SIRT1-mediated autophagy contributes to the protective effects of LM.

## Figures and Tables

**Table 1:** Primer sequences.

Gene	Sequence (5′ → 3′)
H-MMP13	Forward: CCTTGATGCCATTACCAGTCTCC
	Reverse: AAACAGCTCCGCATCAACCTGC
H-SOX9	Forward: AGGAAGCTCGCGGACCAGTAC
	Reverse: GGTGGTCCTTCTTGTGCTGCAC
H-ADAMTS5	Forward: CCTGGTCCAAATGCACTTCAGC
	Reverse: TCGTAGGTCTGTCCTGGGAGTT
H-Collagen II (COL2A1)	Forward: CCTGGCAAAGATGGTGAGACAG
	Reverse: CCTGGTTTTCCACCTTCACCTG
H-Aggrecan (ACAN)	Forward: AGTAGAGGACATCAGCGGGCTT
	Reverse: CCGCTGATGTCCTCTACTCCAG
H-iNOS	Forward: GCTCTACACCTCCAATGTGACC
	Reverse: CTGCCGAGATTTGAGCCTCATG
H-COX2	Forward: CGGTGAAACTCTGGCTAGACAG
	Reverse: GCAAACCGTAGATGCTCAGGGA
H-GAPDH	Forward: GTCTCCTCTGACTTCAACAGCG
	Reverse: ACCACCCTGTTGCTGTAGCCAA

## Data Availability

All datasets generated and analyzed during the present study are available from the corresponding author upon reasonable request.
